# Intermittent selective serotonin reuptake inhibitors for premenstrual syndromes: A systematic review and meta-analysis of randomised trials

**DOI:** 10.1177/02698811221099645

**Published:** 2022-06-10

**Authors:** Thomas J Reilly, Phoebe Wallman, Ivana Clark, Clare-Louise Knox, Michael C Craig, David Taylor

**Affiliations:** 1 Department of Psychosis Studies, Institute of Psychiatry, Psychology and Neuroscience, King’s College London, London, UK; 2 Pharmacy Department, Maudsley Hospital, South London and Maudsley NHS Foundation Trust, London, UK; 3 Department of Psychological Medicine, Institute of Psychiatry, Psychology and Neuroscience, King’s College London, London, UK; 4 Department of Forensic & Neurodevelopmental Sciences, Institute of Psychiatry, Psychology and Neuroscience, King’s College London, London, UK; 5 National Female Hormone Clinic, South London and Maudsley National Health Service Foundation Trust, London, UK; 6 Institute of Pharmaceutical Science, King’s College London, London, UK

**Keywords:** Premenstrual dysphoric disorder, premenstrual syndrome, serotonin uptake inhibitors, randomised controlled trial, meta-analysis

## Abstract

**Background:**

Intermittent (luteal phase) dosing of selective serotonin reuptake inhibitors is one treatment strategy for premenstrual syndromes such as premenstrual dysphoric disorder. This avoids the risk of the antidepressant withdrawal syndrome associated with long-term continuous dosing.

**Aims:**

To compare intermittent dosing to continuous dosing in terms of efficacy and acceptability.

**Methods:**

We searched the Cochrane Central Register of Controlled Trials, MEDLINE, EMBASE, PsycINFO, PubMed and CINAHL for randomised trials of intermittent compared with continuous dosing of selective serotonin reuptake inhibitors in premenstrual syndromes. We extracted response rates, dropout rates and changes in symptom scores. We used random effects meta-analyses to pool study-level data and calculated odds ratio for dichotomous data and standardised mean difference for continuous data. Risk of bias was assessed using the Cochrane risk-of-bias tool. The study was registered with PROSPERO (CRD42020224176).

**Results:**

A total of 1841 references were identified, with eight studies being eligible for analysis, consisting of a total of 460 participants. All included studies provided response rates, six provided dropout rates and five provided symptom scores. There was no statistically significant differences between intermittent and continuous dosing in terms of response rate (odds ratio: 1.0, 95% confidence interval (CI): 0.23–4.31, *I*^2^ = 71%), dropout rate (odds ratio 1.26, 95% CI: 0.39–4.09, *I*^2^ = 33%) or symptom change (standardised mean difference: 0.04, 95% CI: −0.27 to 0.35, *I*^2^ = 39%). All studies had a moderate or high risk of bias.

**Conclusion:**

Since intermittent dosing avoids the potential for withdrawal symptoms, it should be considered more commonly in this patient population.

## Introduction

Antidepressant withdrawal syndrome, particularly after cessation of long-term selective serotonin reuptake inhibitors (SSRIs), has recently been recognised as a significant clinical issue in a position paper by the Royal College of Psychiatrists (2019). The syndrome includes affective symptoms, impaired sleep, sexual dysfunction, disequilibrium, sensory symptoms, gastrointestinal upset and general somatic complaints. Although the incidence is disputed (Davies and Read, 2019; Hengartner, 2019; Jauhar and Hayes, 2019), it is understood to affect a substantial number of patients who are prescribed these medications long-term.

Anti depressants, such as SSRIs, are prescribed to approximately 7.3 million people in England (17% of the adult population) (Public Health England, 2019). SSRIs may be prescribed for various conditions, including premenstrual syndromes (PMS; RCOG, 2017). Several approaches have been taken to define these syndromes, differing mainly with respect to severity of the symptoms required for a diagnosis (RCOG, 2017).

Premenstrual dysphoric disorder (PMDD), previously called late luteal phase dysphoric disorder (LLPDD), is predominately based on psychological symptoms and affects approximately 1–6% of the female population of reproductive age (Cohen et al., 2002; Gehlert et al., 2009). It is defined in the *Diagnostic and Statistical Manual of Mental Disorders, Fifth Edition* (*DSM*-5; American Psychiatric Association, 2013) and International Classification of Diseases: 11th Revision (ICD-11; Reed et al., 2019) by the presence of at least five (out of 11) stipulated symptoms during the luteal phase of the menstrual cycle. The diagnosis requires symptoms to be severe enough to disrupt daily functioning and excludes exacerbation of another psychiatric disorder. PMS also requires subjective reports of dysfunction but is more equally focused on psychological and physical symptoms. The higher prevalence, estimated at 20–30% of women (Yonkers and Simoni, 2018), is mainly due to the limited requirement of only one of six specified mood symptoms and one of four specified physical symptoms. Furthermore, it does not exclude exacerbation of another psychiatric disorder. There is substantial overlap between PMDD and PMS, with a minority of women with PMS also meeting criteria for PMDD (Yonkers and Simoni, 2018).

SSRIs are a first-line treatment for both PMDD and severe PMS (RCOG, 2017), with standardised mean differences (SMD) in the region of 0.65 (0.46–0.84) compared with placebo (Marjoribanks et al., 2013). In contrast to depression, where antidepressant treatment should be continued for 6 months following a first episode and 2 years following a recurrent episode (National Institute for Health and Care Excellence (NICE), 2009), SSRIs for PMDD may be prescribed intermittently. This involves taking the medication daily during the second half of the cycle, known as the luteal phase. Such a dosing regimen eliminates the risk of long-term withdrawal syndrome by avoiding continuous daily use, while short-term withdrawal symptoms have not been found in randomised trials (Yonkers et al., 2005, 2015).

Although luteal phase dosing regimens are recommended as an option by guidelines (RCOG, 2017), it is unclear how this compares with continuous dosing in terms of efficacy or acceptability. Indeed, there are some conflicting reports. A 2008 meta-analysis concluded that intermittent dosing was less effective than continuous (Shah et al., 2008). By contrast, the most recent Cochrane review in 2013 reported that the regimens were equally effective, with the caveat that further research was needed for confirmation (Marjoribanks et al., 2013). Both these analyses crucially included only placebo-controlled studies and therefore excluded trials that directly compared intermittent versus continuous dosing without a placebo-arm. To address this gap in the research to date, we aimed to do a systematic review and meta-analysis of randomised trials comparing intermittent dosing of SSRIs with continuous dosing in PMDD or PMS, examining response rates, dropout rates and symptom reduction.

## Methods

### Search strategy and selection criteria

We included randomised trials in women with either PMDD or PMS which compared intermittent dosing of SSRIs with continuous dosing. Where multiple publications reported results from the same trial, the largest one with most complete data was included. We excluded non-randomised trials. We included studies of at least 2 months. We did not place restrictions based on language and used Google translate for reports that were not in English.

Participants were women diagnosed with PMS by prospective ratings of a validated scale (such as the daily symptoms report; Freeman et al., 1996) or PMDD diagnosed by *DSM* criteria. We excluded studies solely of women who self-report a diagnosis of PMS or PMDD, as this is known to be unreliable (Bosman et al., 2018).

The intervention was a luteal phase dosing, including symptom onset dosing (where the SSRI is taken at first onset of premenstrual symptoms). We excluded semi-intermittent dosing (where the SSRI is given at a higher dose during the luteal phase compared with the follicular phase) (Steiner et al., 2006). The comparator was continuous dosing of an SSRI. We searched for any SSRI: citalopram, escitalopram, fluoxetine, fluvoxamine, paroxetine, sertraline or zimelidine. We also searched for venlafaxine and duloxetine; although these are not traditionally classified as SSRIs, their mechanism of action is similar at lower doses (Harvey et al., 2000).

The following databases were searched from inception until December 2020: MEDLINE, EMBASE, PsycINFO, PubMed and CINAHL. We also searched the Cochrane Central Register of Controlled Trials. References of previous reviews (Marjoribanks et al., 2013; Shah et al., 2008) and included studies were searched. Two authors (T.J.R. and P.W.) screened study abstracts and retrieved potentially relevant full-texts for further examination. Disagreement was resolved by discussion with a third author (I.C.). For studies that were not available or reported insufficient data, we attempted to contact authors by email, with a follow-up request if necessary.

Examples of search keyword terms (adapted for different databases, as shown in the Supplementary Materials) are given below:

- Premenstrual Syndrome OR Premenstrual Dysphoric Disorder OR Late luteal OR PMS OR PMDD OR LLPDD
- Citalopram OR Escitalopram OR Fluoxetine OR Fluvoxamine OR Paroxetine OR Sertraline OR Zimelidine OR Duloxetine OR Venlafaxine
- Random OR Trial

### Data analysis

Two authors (T.J.R. and P.W.) extracted study-level data in duplicate with any disagreement discussed with a third author (I.C.). The following variables of interest were extracted from each study: study setting, sample size, mean age, diagnosis of interest, method of diagnosis, name of SSRI and dosage.

The two pre-specified primary outcome measures were response rate and dropout rate. The secondary outcome measure was SMD of symptom ratings. Response rate was defined using improvement on global scale (such as Clinical Global Impression Scale (Busner and Targum, 2007) or as 50% reduction in symptoms using a continuous measure of symptoms score. Dropout rate was defined as participants leaving the trial early for any reason. For studies reporting symptom scores, the SMD was calculated between the intermittent and continuous dosing groups. Symptom end-scores were extracted in preference to change scores. Where means and standard deviations were not reported, we calculated an effect size based on reported sample size, test statistic and *p* values. We analysed data on an intention-to-treat basis and requested missing data from original study authors. We used Hedges’ *g* as the effect size measure due to potential bias from small sample sizes (Hedges, 2011).

The Cochrane risk-of-bias assessment tool (version 2) (Sterne et al., 2019) was used, independently by two authors (T.J.R. and P.W.), with disagreement resolved by a third author (I.C.).

All analyses were carried out using *R* version 3.6.3, using the ‘meta’ and ‘metafor’ packages, with the ‘dmetar’ package for Egger’s tests. We used a random effects model to pool results across studies. Heterogeneity was assessed using the *I*^2^ statistic. We pre-specified the following subgroup analyses if more than three studies were included: individual SSRI medication, SSRI dosage (low, medium or high), diagnosis for study inclusion (severe PMS or PMDD). Post hoc, where there was doubt about a study’s suitability for inclusion or the data provided, we conducted sensitivity analyses by excluding that study. Small sample bias (publication bias) was assessed visually using funnel plots, with asymmetry quantified using Egger’s test. We used the trim and fill method to correct for asymmetry where this was evident, providing an adjusted-effect size.

We followed PRISMA guidelines (Moher et al., 2009) (see checklist in Supplementary Material) and pre-registered with PROSPERO (CRD42020224176, available from https://www.crd.york.ac.uk/prospero/display_record.php?ID=CRD42020224176). All analyses were carried out after registration, and there were no deviations from the original pre-registration, other than carrying out post hoc sensitivity analyses and subgroup analyses of high and moderate bias.

## Results

**Figure 1.**
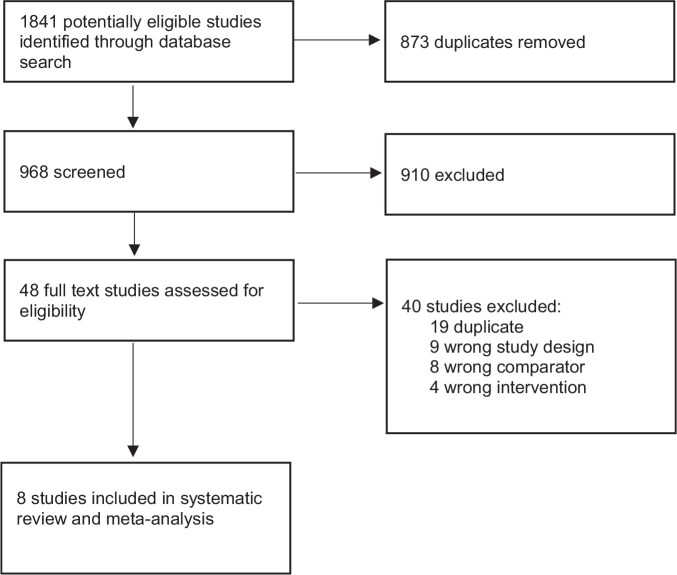
PRISMA flow diagram of study selection.

The search retrieved 1841 records, of which eight met the inclusion criteria, see Figure 1. The characteristics of the included studies are shown in Table 1. In total, 212 participants were randomised to intermittent SSRIs and 248 to continuous SSRIs. Five studies used the diagnosis of PMDD or LLPDD for inclusion, and three used severe PMS. Sertraline and citalopram were each used by three studies, while paroxetine was used by two. All included studies provided response rates, six provided dropout rates and five provided symptom scores.

**Table 1.** Characteristics of included studies.

First author	Year	Setting	Diagnosis	Diagnostic instrument	Treatment length	Dose	SSRI	Outcomes
Alpay and Turhan (2001)	2001	Turkey	PMDD	*DSM*-IV	6 months	50 mg	Sertraline	Response (no recurrence of PMDD symptoms), dropout
Flores-Ramos et al. (2003)	2003	Mexico	PMDD	*DSM*-IV	2 months	20 mg	Citalopram	Response (decrease of 10 points in Moos Menstrual Distress Questionnaire), dropout
Freeman et al. (1999)	1999	USA	Severe PMS	DSR	3 months	50–150 mg	Sertraline	Response (premenstrual DSR score of less than 80), dropout, symptoms (DSR)
Freeman et al. (2002)	2002	USA	Severe PMS	DSR	3 months	20–40 mg	Citalopram	Response (50% decrease in DSR), symptoms (DSR)
Freeman et al. (2004)	2004	USA	Severe PMS	DSR	3 months	50–100 mg	Sertraline	Response (50% reduction in the mean premenstrual DSR), dropout, symptoms (DSR)
Landén et al. (2007)	2007	Sweden	PMDD	*DSM*-IV	3 months	10–20 mg	Paroxetine	Response (very much improved or much improved on the CGI global improvement scale), dropout, symptoms (VAS irritability)
Wikander et al. (1998)	1998	Sweden	LLPDD	*DSM*-III	3 months	10–30 mg	Citalopram	Response (self-reported as much improved or very much improved), dropout, symptoms (VAS irritability)
Wu et al. (2008)	2008	China	PMDD	*DSM*-IV	4 months	20 mg	Paroxetine	Response (CGI Severity 2 or less)

SSRI: selective serotonin reuptake inhibitor; PMDD: premenstrual dysphoric disorder; PMS: premenstrual syndrome; LLPDD: late luteal phase dysphoric disorder; *DSM*: diagnostic and statistical manual; DSR: daily symptom report; CGI: clinical global impression; VAS: Visual Analogue Scale.

**Figure 2.**
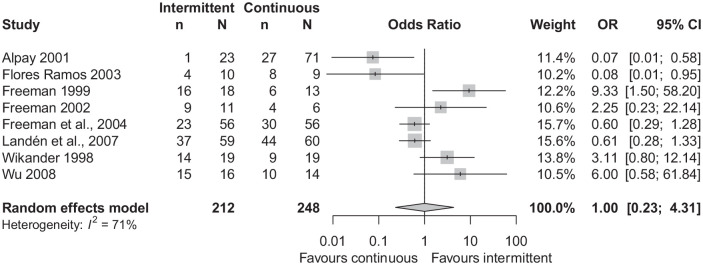
Forest plot of response rates. *n*: number of responders; *N*: total number of participants in the group.

There was no statistically significant difference in response rates between intermittent and continuous dosing (odds ratio (OR): 1.0 (95% CI: 0.23–4.31)), see Figure 2. There was substantial statistical heterogeneity (*I*^2^ = 71%). There were no statistically significant differences in response rates between intermittent and continuous dosing in the following subgroup analyses: five studies with PMDD as the inclusion diagnosis (Supplementary Figure 2), three studies with severe PMS as the inclusion diagnosis (Supplementary Figure 3) and four studies with citalopram as the SSRI (Supplementary Figure 4). Due to concerns about study design, it is questionable whether (Alpay and Turban, 2001) should be included. A sensitivity analysis conducted without this study showed no significant difference in response rates between groups (Supplementary Figure 5). A funnel plot did not show asymmetry (Supplementary Figure 6), and Egger’s test was non-significant (*p* = 0.61).

**Figure 3.**
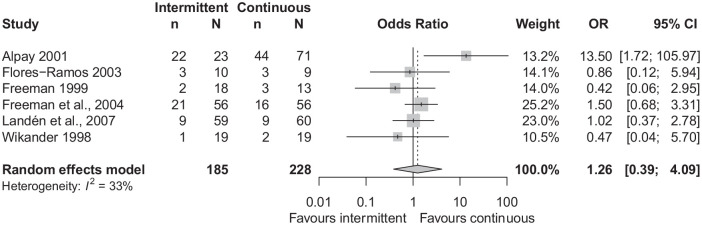
Forest plot of dropout rates. *n*: number of dropouts; *N*: total number of participants in the group.

There was no statistically significant difference in dropout rates between groups (OR: 1.26 (95% CI: 0.39–4.09)), see Figure 3. Statistical heterogeneity was moderate (*I*^2^ = 33%). There were no statistically significant differences in dropout rates in the two subgroup analyses: four studies with PMDD as the inclusion diagnosis (Supplementary Figure 7), three studies with sertraline as the SSRI (Supplementary Figure 8). Since 22 of 23 participants in the intermittent group dropped out, it is questionable whether (Alpay and Turban, 2001) should be included. A sensitivity analysis without this study did not affect the overall result, with no statistically significant differences in dropout rates between groups (Supplementary Figure 9). A funnel plot did not show asymmetry (Supplementary Figure 10), and Egger’s test was non-significant (*p* = 0.91).

**Figure 4.**
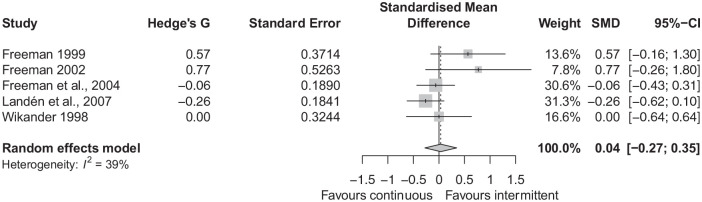
Forest plot of standardised mean difference in symptom scores.

There was no statistically significant difference between groups in terms of change in symptoms scores, with an SMD of 0.04 (95% CI: −0.27 to 0.35), see Figure 4. There was moderate statistical heterogeneity, *I*^2^ = 39%. In the subgroup of three studies with severe PMS as the inclusion diagnosis, there was no difference between groups (Supplementary Figure 11). Excluding Wikander et al. (1998) (whose data we took from a previous Cochrane review, Marjoribanks et al. (2013) again resulted in no difference between groups (Supplementary Figure 12). There was asymmetry noted in the funnel plot (Supplementary Figure 13), Egger’s test was significant (*p* = 0.040), with two small studies (Freeman et al., 1999, 2002), providing large effect sizes in favour of intermittent dosing. Using the trim and fill method (Supplementary Figure 14), the adjusted SMD was −0.130 (95% CI: −0.487 to 0.227), indicating there was no significant difference between groups.

The Cochrane risk-of-bias assessments are summarised in Table 2. Notably, no study was assessed as being low risk of bias, four had some concerns and four had a high risk of bias (elaboration of these concerns are detailed in Supplementary Table 1). Post hoc subgroup analyses of studies grouped by high bias or some concerns of bias are shown in Supplementary Figures 14–17, and none showed statistically significant differences between groups across outcomes.

**Table 2.** Summary of Cochrane risk-of-bias tool for randomised trials (version 2).

Study	Randomisation process	Deviations from intended interventions	Missing outcome data	Measurement of outcome	Selection of reported result	Overall bias
Alpay and Turhan (2001)	High	High	Low	High	Some concerns	High
Flores-Ramos et al. (2003)	Some concerns	Low	Low	Low	High	High
Freeman et al. (1999)	Some concerns	Low	Low	Low	Some concerns	Some concerns
Freeman et al. (2002)	High	Low	Low	Some concerns	Some concerns	High
Freeman et al. (2004)	Low	Low	Low	Low	Some concerns	Some concerns
Landén et al. (2007)	Low	Low	Low	Low	Some concerns	Some concerns
Wikander et al. (1998)	Low	Low	Low	Low	Some concerns	Some concerns
Wu et al. (2008)	High	Low	Low	Low	Some concerns	High

## Discussion

We found no differences between intermittent (luteal phase) and continuous dosing of SSRIs for PMDD or severe PMS in terms of response rates, dropout rates and changes in symptom scores. This finding was consistent across all pre-specified subgroup analyses and post hoc sensitivity analyses.

Although previous meta-analyses have concluded that intermittent dosing of SSRIs is more effective than placebo (Marjoribanks et al., 2013; Shah et al., 2008), this is the first analysis of intermittent compared with continuous SSRI dosing, taking all available evidence into account. Compared with the most recent Cochrane review (Marjoribanks et al., 2013), we were able to include an additional five studies examining response rates and an additional three studies examining symptoms scores. We thus add to the evidence-base that intermittent dosing is as effective as continuous dosing across a range of measures, in support of current guidelines (RCOG, 2017).

Intermittent dosing could be an important treatment option for women who are concerned about long-term withdrawal effects of SSRIs. Since the SSRI is not taken continuously, there will be little or no homeostatic adaption of the serotonergic system to the medication, and therefore, limited risk of receptor downregulation which is thought to underlie SSRI withdrawal symptoms (Horowitz and Taylor, 2019). Evidence from two randomised controlled trials suggests that there are no short-term withdrawal effects associated with intermittent dosing in the context of PMDD (Yonkers et al., 2005, 2015). Our results suggest that the choice of intermittent SSRI dosing would not result in less-effective control of symptoms or dropout from treatment.

The effectiveness of intermittent dosing adds to the body of evidence that, in PMSs, SSRIs have a rapid onset of action. Symptom improvement with fluoxetine is seen within hours, with responsiveness peaking within 2 days (Steinberg et al., 2012). After two cycles of luteal treatment with fluoxetine, symptoms relapse immediately in the third cycle following withdrawal (Pearlstein et al., 2003). These converging clinical data do not support a delayed mechanism of action of SSRIs, as has been argued for depression (Harmer et al., 2009).

An alternative hypothesised mechanism of action in PMDD is through increasing the rate of conversion of the progesterone metabolite 5α-dihydroprogesterone to allopreganolone, a neuroactive steroid which modulates the GABA-A receptor (Hantsoo and Epperson, 2020). This is in keeping with the wider conceptualisation of PMDD as a condition with abnormal sensitivity to allopregnanolone (Hantsoo and Epperson, 2020).

Stigma associated with antidepressants can affect therapeutic adherence and outcome (Castaldelli-Maia et al., 2011). The differences between the action of SSRIs in PMSs and depression may improve the perception of these medications among the PMDD community. Some women with PMDD report feeling ‘dismissed’ with antidepressants (Osborn et al., 2020) and are reluctant to trial medications that are not specific for PMDD (Osborn et al., 2020). Emphasising that SSRIs do not act simply as antidepressants in this condition could improve the acceptability of this treatment option.

There are some important limitations to our review. First, all studies were relatively old, and the most recent was conducted in 2008. As a result, we were unable to gain much of the additional data we requested from study authors. As these studies were conducted in an era when practices such as the pre-registration of protocols were less common, all studies were judged as being moderate to high risk of bias. Of particular concern is that three studies rated as high risk of bias were not double-blind (Alpay and Turhan, 2001; Freeman et al., 2002; Wu et al., 2008). Nevertheless, there is no reason to suggest studies would be biased in a particular direction, either towards intermittent or continuous dosing.

The median study length was 3 months, which is too short to test whether there were differences in the long-term effects of SSRIs. The inclusion of Alpay and Turhan (2001) was questionable; although reported as a randomised design, there was a marked difference in the numbers between the two groups. Similarly, the data used for Wikander et al. (1998) were difficult to interpret as they were not taken from the study manuscript but from a Cochrane review (Marjoribanks et al., 2013). However, excluding these studies did not significantly influence the overall results. The total sample sizes of our meta-analyses are small but do represent the largest comparison of intermittent with continuous dosing to date. Reflecting the sample size, confidence intervals were wide, particularly for subgroup analyses, which contained as few as three studies. Although this indicates uncertainty, none of our pre-specified analysis detected any significant differences between the dosing regimens.

There was substantial heterogeneity in the analysis of response rates. As shown in Figure 2, both Alpay and Turhan (2001) and Flores-Ramos et al. (2003) favoured continuous dosing, Freeman et al. (1999) favoured intermittent dosing, while the other five studies found no effect. As previously mentioned, the inclusion of Alpay and Turhan (2001) is questionable due to concerns about its design. Unusually, only a single participant in this study responded to intermittent dosing. When removed, the heterogeneity reduced to *I*^2^ = 67%, as shown in Supplementary Figure 5. Another source of heterogeneity could be differences in classifying participants as responders. As shown in Table 1, each study used a different definition of response. By contrast, in the symptom ratings studies, only two symptoms rating scales were used across studies (Daily Symptom Report and Visual Analogue Scale Irritability). There was less heterogeneity in the analyses of symptom scores and dropout rates, with both being classified as moderate.

Publication bias was assessed using funnel plots and Egger’s test to detect asymmetry. However, given the total number of studies was less than 10 (the minimum number suggested by the Cochrane handbook Higgins et al., 2019), we had limited power to detect this particular bias. Significant asymmetry was found for studies reporting symptom changes. An adjusted effect size calculated through trim and fill did not find a difference between the dosing regimens for this outcome. If publication bias for the primary outcomes has gone undetected, small studies showing no differences between groups would be more likely to go unpublished, which would not change our overall results.

Finally, although we did not find statistically significant differences between the intermittent and continuous dosing regimens, this does not necessarily imply that clinically meaningful differences between the groups can be ruled out. There is a wide range of values included in the confidence intervals for the outcomes of response and dropout. Post hoc, using the two one-sided tests procedure to reject a smallest effect size of interest of *d* = 0.3, the regimes were equivalent only in the outcome of reducing symptoms (Lakens et al., 2018).

## Conclusion

We found no differences between intermittent and continuous dosing of SSRIs for PMDD or severe PMS. This was the case for response rates, dropout rates and symptom reduction, over the short-term. These findings are of interest because intermittent dosing would mitigate against the risk of withdrawal symptoms that occur following long-term continuous use of SSRI medications.

## Supplemental Material

sj-docx-1-jop-10.1177_02698811221099645 – Supplemental material for Intermittent selective serotonin reuptake inhibitors for premenstrual syndromes: A systematic review and meta-analysis of randomised trials Click here for additional data file. Supplemental material, sj-docx-1-jop-10.1177_02698811221099645 for Intermittent selective serotonin reuptake inhibitors for premenstrual syndromes: A systematic review and meta-analysis of randomised trials by Thomas J Reilly, Phoebe Wallman, Ivana Clark, Clare-Louise Knox, Michael C Craig and David Taylor in Journal of Psychopharmacology
