# Supplementary material for: Intermittent selective serotonin reuptake inhibitors for premenstrual syndromes: A systematic review and meta-analysis of randomised trials
Source: J Psychopharmacol. 2022 Jun 10;37(3):261–7. doi: 10.1177/02698811221099645 (PMC10074750; doi:10.1177/02698811221099645)
Supplement: sj-docx-1-jop-10.1177_02698811221099645 – Supplemental material for Intermittent selective serotonin reuptake inhibitors for premenstrual syndromes: A systematic review and meta-analysis of randomised trials [file sj-docx-1-jop-10.1177_02698811221099645.docx]

**Supplementary Material**

**Intermittent selective serotonin reuptake inhibitors for premenstrual syndromes: Systematic review and meta-analysis of randomised trials**

Thomas J Reilly, Phoebe Wallman, Ivana Clark, Clare-Louise Knox, Michael C Craig, David Taylor

**Table of Contents**

| Specific search strings | 28 |
| --- | --- |
| List of studies excluded at full-text screening | 39 |
| PRISMA checklist | 47 |
| Supplementary figures  Supplementary table | 50  58 |
| Study Protocol | 59 |

**Specific search strings**

Database: Ovid MEDLINE(R) ALL <1946 to December 29, 2020>

Search Strategy:

--------------------------------------------------------------------------------

1 exp Premenstrual Syndrome/ or "Premenstrual syndrom*".mp. (4677)

2 "PMS".mp. (6278)

3 exp Premenstrual Dysphoric Disorder/ or "Premenstrual dysphoric".mp. (948)

4 "PMDD".mp. (634)

5 "Late Luteal Phase Dysphoric".mp. (74)

6 "LLPDD".mp. (30)

7 exp Serotonin Uptake Inhibitors/ or "Selective serotonin reuptake inhibitor*".mp. (47086)

8 "SSRI*".mp. (10430)

9 Citalopram.mp. or exp Citalopram/ (7118)

10 escitalopram.mp. (2628)

11 fluoxetine.mp. or exp Fluoxetine/ (14347)

12 fluvoxamine.mp. or exp Fluvoxamine/ (2994)

13 paroxetine.mp. or exp Paroxetine/ (6432)

14 exp Sertraline/ or setraline.mp. (3093)

15 venlafaxine.mp. or exp Venlafaxine Hydrochloride/ (4525)

16 duloxetine.mp. or exp Duloxetine Hydrochloride/ (2785)

17 zimelidine.mp. or exp Zimeldine/ (633)

18 Random*.mp. (1425329)

19 Trial*.mp. (1767210)

20 "premenstrual tension*".mp. (682)

21 1 or 2 or 3 or 4 or 5 or 6 or 20 (10037)

22 7 or 8 or 9 or 10 or 11 or 12 or 13 or 14 or 15 or 16 or 17 (58040)

23 18 or 19 (2324137)

24 21 and 22 and 23 (213)

***************************

Database: Embase <1974 to 2020 Week 52>

Search Strategy:

--------------------------------------------------------------------------------

1 exp premenstrual syndrome/ or "Premenstrual syndrom*".mp. (6131)

2 PMS.mp. (8799)

3 exp premenstrual dysphoric disorder/ or "premenstrual dysphoric".mp. (1692)

4 PMDD.mp. (882)

5 "Late luteal phase dysphoric".mp. (101)

6 LLPD.mp. (9)

7 exp serotonin uptake inhibitor/ or "Selective serotonin reuptake inhibitor*".mp. (272656)

8 SSRI*.mp. (18013)

9 citalopram.mp. or exp citalopram/ (23510)

10 escitalopram.mp. or exp escitalopram/ (12645)

11 exp fluoxetine/ or fluoxetine.mp. (48604)

12 exp fluvoxamine/ or fluvoxamine.mp. (14236)

13 paroxetine.mp. or exp paroxetine/ (28428)

14 sertraline.mp. or exp sertraline/ (26868)

15 venlafaxine.mp. or exp venlafaxine/ (21973)

16 duloxetine.mp. or exp duloxetine/ (11512)

17 zimelidine.mp. or exp zimeldine/ (1820)

18 random*.mp. (1843011)

19 trial*.mp. (2561180)

20 "premenstrual tension*".mp. (374)

21 1 or 2 or 3 or 4 or 5 or 6 or 20 (14150)

22 7 or 8 or 9 or 10 or 11 or 12 or 13 or 14 or 15 or 16 or 17 (275463)

23 18 or 19 (3282158)

24 21 and 22 and 23 (578)

***************************

Database: APA PsycInfo <1806 to December Week 3 2020>

Search Strategy:

--------------------------------------------------------------------------------

1 "premenstrual syndrom*".mp. (2196)

2 PMS.mp. or exp Premenstrual Syndrome/ (2174)

3 exp Premenstrual Dysphoric Disorder/ or "premenstrual dysphoric".mp. (759)

4 PMDD.mp. (492)

5 "late luteal phase dysphoric".mp. (109)

6 LLPDD.mp. (45)

7 exp Serotonin Reuptake Inhibitors/ or "selective serotonin reuptake inhibitor*".mp. (15364)

8 SSRI*.mp. (6713)

9 exp Citalopram/ or citalopram.mp. (3557)

10 escitalopram.mp. (1663)

11 fluoxetine.mp. or exp Fluoxetine/ (7458)

12 exp Fluvoxamine/ or fluvoxamine.mp. (1773)

13 exp Paroxetine/ or paroxetine.mp. (3621)

14 sertraline.mp. or exp Sertraline/ (3208)

15 venlafaxine.mp. or exp Venlafaxine/ (2537)

16 duloxetine.mp. (1086)

17 exp Zimeldine/ or zimelidine.mp. (178)

18 Random*.mp. (217946)

19 Trial*.mp. (190877)

20 "Premenstrual tension*".mp. (184)

21 1 or 2 or 3 or 4 or 5 or 6 or 20 (3061)

22 7 or 8 or 9 or 10 or 11 or 12 or 13 or 14 or 15 or 16 or 17 (25028)

23 18 or 19 (335346)

24 21 and 22 and 23 (103)

***************************

Database: PubMed, 31^st^ December 2020

Search Strategy:

--------------------------------------------------------------------------------

Search:((premenstrual syndrom* OR PMS OR premenstrual tension*) OR (premenstrual dysphoric OR PMDD) OR (late luteal phase dysphoric OR LLPDD)) AND ((selective serotonin reuptake inhibitor* OR SSRI*) OR (citalopram OR escitalopram OR fluoxetine OR fluvoxamine OR paroxetine OR sertraline OR venlafaxine OR duloxetine OR zimelidine)) AND (random* OR trial*)

***************************

Database: CINAHL 31^st^ December 2020

Search Strategy:

--------------------------------------------------------------------------------

1 (MH “Premenstrual Dysphoric Disorder”) OR (MH “Premenstrual Syndrome+”) OR ““premenstrual syndrome*” OR PMS OR “premenstrual tension*” OR “premenstrual dysphoric” OR PMDD OR “late luteal phase dysphoric” OR LLPDD” (1,838)

2 (MH “Serotonin Uptake Inhibitor+”) OR (MH “Citalopram”) OR (MH “Fluvoxamine Maleate”) OR (MH “Fluoxetine+”) OR (MH “Venlafaxine+”) OR (MH “Sertraline Hydrochloride”) OR (MH “Paroxetine”) OR (MH “Duloxetine Hydrochloride”) OR ““selective serotonin reuptake inhibitor*” OR SSRI* OR citalopram OR escitalopram OR fluoxetine OR fluvoxamine OR paroxetine OR sertraline OR venlafaxine OR duloxetine OR zimelidine” (11,785)

3 1 and 2 (187)

4 ““random*” OR “trial*”” (240,583)

5 3 and 4 (20)

***************************

Web of science 31^st^ December 2020

Search Strategy:

--------------------------------------------------------------------------------

1. ALL = (“premenstrual syndrome*” OR PMS OR “premenstrual tension*” OR “premenstrual dysphoric*” OR PMDD OR “late luteal phase dysphoric” OR LLPDD) (19,183)
2. ALL = (“selective serotonin reuptake inhibitor*” OR SSRI* OR citalopram OR escitalopram OR fluoxetine OR fluvoxamine OR paroxetine OR sertraline OR venlafaxine OR duloxetine OR zimelidine) (52,139)
3. 1 and 2 (532)
4. All = (random* OR trial*) (2,970,414)
5. 3 and 4 (272)

***************************

Cochrane Central Register of Controlled Trials

Date Run: 31/12/2020

ID Search Hits

1 ("premenstrual syndrom*" OR PMS):ti,ab,kw (Word variations have been searched)

2 MeSH descriptor: [Premenstrual Syndrome] explode all trees

3 ("premenstrual tension*"):ti,ab,kw

4 ("premenstrual dysphoric" OR PMDD):ti,ab,kw (Word variations have been searched)

5 MeSH descriptor: [Premenstrual Dysphoric Disorder] explode all trees

6 ("Late luteal phase dysphoric" OR "LLPDD"):ti,ab,kw (Word variations have been searched)

7 #1 OR #2 OR #3 OR #4 OR #5 OR #6

8 ("selective serotonin reuptake inhibitor*" OR SSRI*):ti,ab,kw (Word variations have been searched)

9 MeSH descriptor: [Serotonin Uptake Inhibitors] explode all trees

10 (citalopram OR escitalopram OR fluoxetine OR fluvoxamine OR paroxetine OR sertraline OR venlafaxine OR duloxetine OR zimelidine):ti,ab,kw (Word variations have been searched)

11 MeSH descriptor: [Citalopram] explode all trees

12 MeSH descriptor: [Fluvoxamine] explode all trees

13 MeSH descriptor: [Fluoxetine] explode all trees

14 MeSH descriptor: [Paroxetine] explode all trees

15 MeSH descriptor: [Sertraline] explode all trees

16 MeSH descriptor: [Venlafaxine Hydrochloride] explode all trees

17 MeSH descriptor: [Duloxetine Hydrochloride] explode all trees

18 MeSH descriptor: [Zimeldine] explode all trees

19 #8 OR #9 OR #10 OR #11 OR #12 OR #13 OR #14 OR #15 OR #16 OR #17 OR #18

20 #7 AND #19

**List of studies excluded at full-text screening**

#397 Yonkers, K. A.; Halbreich, U.; Freeman, E.; Brown, C.; Pearlstein, T. Sertraline in the treatment of premenstrual dysphoric disorder. Psychopharmacology bulletin;32(1):41-6

Wrong intervention

#1119 Steiner, M.; Hirschberg, A. L.; Bergeron, R.; Holland, F.; Gee, M. D.; Van Erp, E. Luteal phase dosing with paroxetine controlled release (CR) in the treatment of premenstrual dysphoric disorder. American Journal of Obstetrics & Gynecology ;193(2):352-60

Wrong comparator

#374 Yonkers, K. A.; Pearlstein, T.; Fayyad, R.; Gillespie, J. A.

Luteal phase treatment of premenstrual dysphoric disorder improves symptoms that continue into the postmenstrual phase. Journal of affective disorders ;85(3):317-21

Wrong study design

#465 Nilsson, M; Judge, R; Brown, E; Schuler, C. Fluoxetine's efficacy in improving mood, physical and social impairment symptoms associated with PMDD. International Journal of Gynecology & Obstetrics 2000;70():A96-A96

Wrong intervention

#541 Miner, C. M.; Brown, E.; Dillon, J. Efficacy of intermittent fluoxetine dosing on the physical symptoms of PMDD. 2001 annual meeting of the american psychiatric association; 2001 may 5-10; new orleans; LA, USA 2001;():

Duplicate

#1134. Steiner, M.; Brown, E.; Dillon, J. Fluoxetine reduces food cravings during the luteal phase in women with PMDD 2001 annual meeting of the american psychiatric association; 2001 may 5-10; new orleans; LA, USA 2001;():

Duplicate

#464 Nilsson, M.; Judge, R.; Brown, E.; Schuler, C. Fluoxetine's efficacy in improving mood, physical and social impairment symptoms associated with PMDD

XVI FIGO world congress of o & g 2000;Abstract book 1():96

Duplicate

#1028 Gee, M; Bellew, K; Holland, F; Van Erp, E; Perera, P; McCafferty, J. Luteal phase dosing of paroxetine controlled-release is effective in treating premenstrual dysphoric disorder (PMDD). American Psychological Association Meeting 2003;():

Duplicate

#1473 Cohen Lee, S.; Miner Cherri, M.; Brown, Eileen; Dillon, Julia. Efficacy of intermittent fluoxetine dosing in pmdd. 155th annual meeting of the american psychiatric association; 2002 may 18-23rd; philadelphia, PA, USA 2002;():

Duplicate

#857 Ramos, M. F.; Uribe, M. O.; Sotres, J. C. Continous versus intermittent treatment with citalpran in premenstrual dysphoric disorder. Salud Mental Jun 2003;26(3):37-45

Duplicate

#1339 - Flores Ramos, M.; Ontiveros Uribe, M.; Cortes Sotres, J. Continuous versus intermittent treatment with citalopram in premenstrual dysphoric disorder. [Spanish] Salud Mental June 2003;26(3):37-45 Duplicate

#1545 - Halbreich, U.; Bergeron, R.; Stout, A.; Freeman, E.; Yonkers, K.; Pearlstein, T.; Harrison, W. Intermittent luteal phase dosing of sertraline is effective in premenstrual dysphoric disorder. 153rd annual meeting of the american psychiatric association chicago, illionois, USA may 13th-18th 2000 2000;(): Duplicate

#1013 – GlaxoSmithKline. A Placebo-Controlled Study to Investigate the Efficacy of Intermittent and Continuous Treatment With Paroxetine in Patients With Premenstrual Dysphoric Disorder (PMDD) GSK - clinical study register [www.gsk-clinicalstudyregister.com] 2000

Duplicate

#771 - Rohde, A.; Klemme, A. Premenstrual dysphoric disorder as the most severe form of the premenstrual syndrome. Geburtshilfe und Frauenheilkunde Apr 2002;62():17-25

Duplicate

#772 - Rohde, A.; Klemme, A. Premenstrual dysphoric disorder as the most severe form of the premenstrual syndrome. Geburtshilfe und Frauenheilkunde 2002;62(SUPPL. 1):17-25

Wrong study design

#1431 - Crnobaric, C.; Jasovic-Gasic, M.; Milovanovic, S.; Miljevic, C. Treatment of premenstrual dysphoric disorder with fluoxetine during the luteal phase

9th congress of the association of european psychiatrists. Copenhagen, denmark. 20-24th september 1998. 1998;():

Wrong comparator

#630 Kornstein, S. G.; Smith, K. C. Antidepressant treatment of premenstrual syndrome and premenstrual dysphoric disorder. Primary Psychiatry December 2004;11(12):53-57

Wrong study design

#195 Stone, A. B.; Pearlstein, T. B.; Brown, W. A. Fluoxetine in the treatment of late luteal phase dysphoric disorder. Journal of clinical psychiatry ;52(7):290-3

Wrong intervention

#1329 Freeman , EW; Rickels, K; Sondheimer , SJ. Comparison of serotonergic and noradrenergic antidepressant medications in treatment of premenstrual syndrome (PMS). XXIst Collegium Internationale Neuro-psychopharmacologicum 1998;():

Wrong study design

#773 - Rohde, A. Use of antidepressants in premenstrual syndrome and in the climacteric. [German] Gynakologische Praxis Third Quarter 2004;28(3):436-437

Wrong study design

#1512 Halbreich, U.; Smoller, J. W. Intermittent luteal phase sertraline treatment of dysphoric premenstrual syndrome. Journal of clinical psychiatry ;58(9):399-402

Wrong comparator

#1537 Halbreich, U.; Bergeron, R.; Yonkers, K. A.; Freeman, E.; Stout, A. L.; Cohen, L. Efficacy of intermittent, luteal phase sertraline treatment of premenstrual dysphoric disorder. Obstetrics & Gynecology ;100(6):1219-29.

Wrong comparator

#56 Wohlfarth, A. Premenstrual dysphoria. [German] Pharmazeutische Zeitung 26 Oct 2006;151(43):31. Wrong study design

#1474 Cohen, L; Miner, C; Brown, E; Dillon, J. Efficacy of intermittent fluoxetine dosing in premenstrual dysphoric disorder (PMDD) European neuropsychopharmacology 2001;(11):S210

Wrong comparator

#1349 Fernandes, O.; Por, C. P.; Evans, M. F. Is sertraline an effective therapy for premenstrual dysphoric disorder? Canadian family physician 1998;44():765‐767

Wrong study design

#1106 Steiner, M.; Korzekwa, M.; Lamont, J.; Wilkins, A. Intermittent fluoxetine dosing in the treatment of women with premenstrual dysphoria. Psychopharmacology bulletin ;33(4):771-4

Wrong study design

#1546 Halbreich, U; Bergeron, R; Freeman, E; Stout, A; Cohen, L. Intermittent luteal phase dosing of sertraline effective in PMDD. Int J Neuropsychopharmacol 2000;3(suppl 1):S248

Wrong comparator

#1194 Menkes, D. B.; Taghavi, E.; Mason, P. A.; Spears, G. F.; Howard, R. C. Fluoxetine treatment of severe premenstrual syndrome

BMJ ;305(6849):346-7

Wrong intervention

#584 Landen, M; Ysander, C; Sorvik, K; Nissbrandt, H; Allgulander, C; Hunter, B; Eriksson, E. A placebo-controlled study of the efficacy of intermittent and continuous treatment with paroxetine for premenstrual dysphoric disorder (PMDD)

European neuropsychopharmacology 2001;(11):S308-S309

Duplicate

#1525 - Halbreich, U.; Kahn, L. S. Treatment of premenstrual dysphoric disorder with luteal phase dosing of sertraline. Expert Opinion on Pharmacotherapy Nov 2003;4(11):2065-2078

Wrong study design

#631 Kornstein, S. G.; Pearlstein, T. B.; Fayyad, R.; Farfel, G. M.; Gillespie, J. A. Low-dose sertraline in the treatment of moderate-to-severe premenstrual syndrome: efficacy of 3 dosing strategies. Journal of clinical psychiatry ;67(10):1624-32

Wrong comparator

#542 Miner Cherri, M.; Brown, Eileen. Efficacy of intermittent fluoxetine dosing on the physical symptoms of pmdd. 155th annual meeting of the american psychiatric association; 2002 may 18-23rd; philadelphia, PA, USA 2002;():

Wrong comparator

#1170 Sondheimer Stephen, J.; Espana Beatriz, Garcia. Sertraline in premenstrual dysphoric disorder patients on oral contraceptives. 155th annual meeting of the american psychiatric association; 2002 may 18-23rd; philadelphia, PA, USA 2002;():

Duplicate

#640 Kornstein, S; Gillespie, J. Double blind placebo controlled study of sertraline in premenstrual syndrome 155th Annual Meeting of the American Psychiatric Association 2002

Duplicate

#588 Landen, M; Sorvik, K; Ysander, C; Allgulander, C; Nissbrandt, H; Gezelius, B; Eriksson, E. A placebo-controlled trial exploring the efficacy of paroxetine for the treatment of premenstrual dysphoria. NORDIC JOURNAL OF PSYCHIATRY 2001;55(2):95-95

Duplicate

#1027 Gee, M.; Bellew, K. M.; Holland, F. J.; Van Erp, E.; Perera, P.; McCafferty, J. P. Luteal phase dosing of paroxetine controlled release is effective in treating PMDD

156th annual meeting of the american psychiatric association, may 17-22, san francisco CA 2003

Duplicate

#1461 Cohen, L. S.; Miner, C. M.; Brown, E.; Dillon, J. Efficacy of intermittent fluoxetine dosing in PMDD. 2001 annual meeting of the american psychiatric association. Duplicate

#1005 GlaxoSmithKline. A Placebo- Controlled Study to Investigate the Efficacy of Intermittent and Continuous Treatment With Paroxetine in Patients With Premenstrual Dysphoric Disorder ( PMDD) ‏ 2007

Duplicate

#1332 Freeman, E.; Sondheimer, S.; Garcia-Espana, B. Sertraline in premenstrual dysphoric disorder patients on oral contraceptives. 153rd annual meeting of the american psychiatric association chicago, illionois, USA may 13th-18th 2000

Duplicate

#589 Landen, M.; Sorvik, K.; Ysander, C.; Allgulander, C.; Nissbrandt, B.; Gezelius, B.; Eriksson, E. A placebo-controlled trial exploring the efficacy of paroxetine in PMDD. 155th annual meeting of the american psychiatric association; 2002 may 18-23; philadelphia, PA 2002

**PRISMA checklist**

| **Section and Topic** | **Item #** | **Checklist item** | **Location where item is reported** |
| --- | --- | --- | --- |
| **TITLE** | | |  |
| Title | 1 | Identify the report as a systematic review. | 2 |
| **ABSTRACT** | | |  |
| Abstract | 2 | See the PRISMA 2020 for Abstracts checklist. | 3 |
| **INTRODUCTION** | | |  |
| Rationale | 3 | Describe the rationale for the review in the context of existing knowledge. | 5-6 |
| Objectives | 4 | Provide an explicit statement of the objective(s) or question(s) the review addresses. | 7 |
| **METHODS** | | |  |
| Eligibility criteria | 5 | Specify the inclusion and exclusion criteria for the review and how studies were grouped for the syntheses. | 7 |
| Information sources | 6 | Specify all databases, registers, websites, organisations, reference lists and other sources searched or consulted to identify studies. Specify the date when each source was last searched or consulted. | 8 |
| Search strategy | 7 | Present the full search strategies for all databases, registers and websites, including any filters and limits used. | 28-38 |
| Selection process | 8 | Specify the methods used to decide whether a study met the inclusion criteria of the review, including how many reviewers screened each record and each report retrieved, whether they worked independently, and if applicable, details of automation tools used in the process. | 8 |
| Data collection process | 9 | Specify the methods used to collect data from reports, including how many reviewers collected data from each report, whether they worked independently, any processes for obtaining or confirming data from study investigators, and if applicable, details of automation tools used in the process. | 9 |
| Data items | 10a | List and define all outcomes for which data were sought. Specify whether all results that were compatible with each outcome domain in each study were sought (e.g. for all measures, time points, analyses), and if not, the methods used to decide which results to collect. | 9 |
|  | 10b | List and define all other variables for which data were sought (e.g. participant and intervention characteristics, funding sources). Describe any assumptions made about any missing or unclear information. | 9 |
| Study risk of bias assessment | 11 | Specify the methods used to assess risk of bias in the included studies, including details of the tool(s) used, how many reviewers assessed each study and whether they worked independently, and if applicable, details of automation tools used in the process. | 9 |
| Effect measures | 12 | Specify for each outcome the effect measure(s) (e.g. risk ratio, mean difference) used in the synthesis or presentation of results. | 9 |
| Synthesis methods | 13a | Describe the processes used to decide which studies were eligible for each synthesis (e.g. tabulating the study intervention characteristics and comparing against the planned groups for each synthesis (item #5)). | 10 |
|  | 13b | Describe any methods required to prepare the data for presentation or synthesis, such as handling of missing summary statistics, or data conversions. | 10 |
|  | 13c | Describe any methods used to tabulate or visually display results of individual studies and syntheses. | 10 |
|  | 13d | Describe any methods used to synthesize results and provide a rationale for the choice(s). If meta-analysis was performed, describe the model(s), method(s) to identify the presence and extent of statistical heterogeneity, and software package(s) used. | 9-10 |
|  | 13e | Describe any methods used to explore possible causes of heterogeneity among study results (e.g. subgroup analysis, meta-regression). | 10 |
|  | 13f | Describe any sensitivity analyses conducted to assess robustness of the synthesized results. | 10 |
| Reporting bias assessment | 14 | Describe any methods used to assess risk of bias due to missing results in a synthesis (arising from reporting biases). | 10 |
| Certainty assessment | 15 | Describe any methods used to assess certainty (or confidence) in the body of evidence for an outcome. | 9 |
| **RESULTS** | | |  |
| Study selection | 16a | Describe the results of the search and selection process, from the number of records identified in the search to the number of studies included in the review, ideally using a flow diagram. | Figure 1 |
|  | 16b | Cite studies that might appear to meet the inclusion criteria, but which were excluded, and explain why they were excluded. | 39-46 |
| Study characteristics | 17 | Cite each included study and present its characteristics. | Table 1 |
| Risk of bias in studies | 18 | Present assessments of risk of bias for each included study. | Table 2 |
| Results of individual studies | 19 | For all outcomes, present, for each study: (a) summary statistics for each group (where appropriate) and (b) an effect estimate and its precision (e.g. confidence/credible interval), ideally using structured tables or plots. | Figures 2-4 |
| Results of syntheses | 20a | For each synthesis, briefly summarise the characteristics and risk of bias among contributing studies. | 11-14 |
|  | 20b | Present results of all statistical syntheses conducted. If meta-analysis was done, present for each the summary estimate and its precision (e.g. confidence/credible interval) and measures of statistical heterogeneity. If comparing groups, describe the direction of the effect. | Figures 2-4, 50-57 |
|  | 20c | Present results of all investigations of possible causes of heterogeneity among study results. | 50, 52-54, 57 |
|  | 20d | Present results of all sensitivity analyses conducted to assess the robustness of the synthesized results. | 51, 53, 55 |
| Reporting biases | 21 | Present assessments of risk of bias due to missing results (arising from reporting biases) for each synthesis assessed. | 52, 54-66 |
| Certainty of evidence | 22 | Present assessments of certainty (or confidence) in the body of evidence for each outcome assessed. | Figures 2-4 |
| **DISCUSSION** | | |  |
| Discussion | 23a | Provide a general interpretation of the results in the context of other evidence. | 14-16 |
|  | 23b | Discuss any limitations of the evidence included in the review. | 16-18 |
|  | 23c | Discuss any limitations of the review processes used. | 16-18 |
|  | 23d | Discuss implications of the results for practice, policy, and future research. | 16 |
| **OTHER INFORMATION** | | |  |
| Registration and protocol | 24a | Provide registration information for the review, including register name and registration number, or state that the review was not registered. | 10 |
|  | 24b | Indicate where the review protocol can be accessed, or state that a protocol was not prepared. | 59 |
|  | 24c | Describe and explain any amendments to information provided at registration or in the protocol. | N/A |
| Support | 25 | Describe sources of financial or non-financial support for the review, and the role of the funders or sponsors in the review. | 2 |
| Competing interests | 26 | Declare any competing interests of review authors. | 19 |
| Availability of data, code and other materials | 27 | Report which of the following are publicly available and where they can be found: template data collection forms; data extracted from included studies; data used for all analyses; analytic code; any other materials used in the review. | N/A |

**Supplementary figures**

Supplementary Figure 1. Forest plot of response rates in studies using PMDD as inclusion diagnosis

Supplementary Figure 2. Forest plot of response rates in studies using PMS as diagnosis of inclusion

Supplementary Figure 3. Forest plot of response rates in studies using Citalopram

Supplementary Figure 4. Forest plot of response rates, excluding Alpay.and Turhan 2001

Supplementary Figure 5. Funnel plot for response rates

Supplementary Figure 6. Forest plots of dropout rates in studies using PMDD as the diagnosis of inclusion

Supplementary Figure 7. Forest plots of dropout rates in studies using Sertraline

Supplementary Figure 8. Forest plot of dropout rates excluding Alpay and Turhan. 2001

Supplementary Figure 9. Funnel plot of dropout rates

Supplementary Figure 10. Forest plot of standardised mean difference in symptom scores in studies using PMS as the diagnosis of inclusion

Supplementary Figure 11. Forest plot of standardised mean difference in symptom scores, excluding Wikander at al. 1998

Supplementary Figure 12. Funnel plot of symptom changes

Supplementary Figure 13. Funnel plot of symptom changes including trim and fill imputed studies

Supplementary Figure 14. Forest plot of response rates in studies with some concerns of bias

**

Supplementary Figure 15. Forest plot of response rates in studies with high risk of bias

**

Supplementary Figure 16. Forest plot of dropout rates in studies with some concerns of bias

Supplementary Figure 17. Forest plot of standardised mean difference in symptom scores in studies with some concerns of bias

**Supplementary table**

*Supplementary Table 1. Details of bias concerns*

| Study | Domain | Elaboration | Outcome |
| --- | --- | --- | --- |
| Alpay 2001 | Randomisation process | No information on the randomisation process or whether it was concealed until participants were enrolled and assigned to interventions  The group numbers suggested a problem with the randomisation process | High risk |
|  | Deviations from the intended interventions | Deviation from the intended intervention indicating awareness of assigned intervention  Participants moved into continuous group from intermittent group were analysed as continuous | High risk |
|  | Measurement of the outcome | Unclear how PMDD complaints and adverse effects were measured | High risk |
|  | Selection of the reported result | No pre-specified analysis plan | Some concerns |
| Flores-Ramos 2003 | Randomisation process | No information on whether the allocation sequence was concealed until participants were enrolled and assigned to interventions | Some concerns |
|  | Selection of the reported result | No pre-specified analysis plan  The result assessed is likely to have been selected from multiple eligible outcome measurements within the outcome domain | High risk |
| Freeman 1999 | Randomisation process | No information on the randomisation process or whether it was concealed until participants were enrolled and assigned to interventions | Some concerns |
|  | Selection of the reported result | No pre-specified analysis plan | Some concerns |
| Freeman 2002 | Randomisation process | Open-label study  HAM-D baseline scores suggested a problem with the randomisation process | High risk |
|  | Measurement of the outcome | Assessors aware of the intervention received by study participants and the primary focus of the study was on intermittent dosing | Some concerns |
|  | Selection of the reported result | No pre-specified analysis plan | Some concerns |
| Freeman 2004 | Selection of the reported result | No pre-specified analysis plan | Some concerns |
| Landen 2007 | Selection of the reported result | No pre-specified analysis plan | Some concerns |
| Wikander 1998 | Selection of the reported result | No pre-specified analysis plan | Some concerns |
| Wu 2008 | Randomisation process | No information on the randomisation process  Open-label study | High risk |
|  | Selection of the reported result | No pre-specified analysis plan | Some concerns |

**Study protocol**

**INTERMITTENT SELECTIVE SEROTONIN REUPTAKE INHIBITORS FOR PREMENSTRUAL DYSPHORIC DISORDER: PROTOCOL FOR SYSTEMATIC REVIEW AND META-ANALYSIS**

Authors: Thomas Reilly, Ivana Clark, Phoebe Wallman, David Taylor

Last edited: 02/12/2020

**INTRODUCTION**

Antidepressant withdrawal syndrome has recently been recognised as a significant clinical issue in a position paper by the Royal College of Psychiatrists.^1^ A variety of problems may arise across a number of domains including affective symptoms, impaired sleep, sexual dysfunction, disequilibrium, sensory symptoms, gastrointestinal upset and general somatic complaints. The incidence of this syndrome is disputed^2–4^ but may affect a substantial number of patients who are prescribed these medications long-term.

Antidepressants are prescribed for approximately 7.3 million people in England (17% of the adult population).^5^ In addition to depression, antidepressants may be prescribed for various other conditions, including Premenstrual Syndrome (PMS) and Premenstrual Dysphoric Disorder (PMDD).^6^ PMS, defined by significant emotional and physical symptoms arising during the premenstrual phase of the menstrual cycle, affects approximately 20-30% of women.^7^ PMDD has newly been included in DSM-5^8^ and ICD-11^9^, is also defined by the timing of dysphoric symptoms during the premenstrual phase and is associated with significant functional impairment. PMDD is considered a severe form of PMS and affects approximately 1-6%^10,11^ of women during reproductive years.

Selective Serotonin Reuptake Inhibitors (SSRIs) are a first-line treatment for both PMDD and severe PMS.^6^ In contrast to depression, where antidepressant treatment should be continued for six months following a first episode and two years following a recurrent episode,^12^ SSRIs for PMDD can be prescribed intermittently. This generally involves taking the medication daily during the second half of the cycle, known as the luteal phase. Such a dosing regimen eliminates the risk of long-term withdrawal syndrome, while randomised trials do not show any evidence of shorter-term withdrawal symptoms.^13^

Although intermittent dosing regimens are recommended in guidelines,^6^ it is not clear how this compares with continuous dosing in terms of efficacy or acceptability. Indeed, there are some conflicting reports. A 2008 meta-analysis concluded that intermittent dosing was less effective than continuous.^14^ By contrast the most recent Cochrane review in 2013 reported that both regimes were equally effective, with the caveat that further research was needed for confirmation.^15^ Both these analyses crucially included only placebo-controlled studies and therefore excluded trials that directly compared intermittent versus continuous dosing without a placebo-arm. To address this previous limitation, we will systematically review the evidence for intermittent dosing of SSRIs in PMDD and PMS in comparison to continuous dosing.

**RESEARCH QUESTION**

Is intermittent dosing of SSRIs in women with PMDD or PMS effective and acceptable compared with SSRIs given continuously.

**METHODS**

We will follow PRISMA guidelines in reporting this study.^16^

**Selection Criteria**

We will include published or unpublished randomised trials in women with either PMDD (previously called Late Luteal Phase Dysphoric Disorder) or PMS which compare intermittent dosing of SSRIs to continuous dosing. Where multiple publications report results from the same trial, the largest one with most complete data will be included. We will exclude non-randomised trials. We will use the first phase only from cross-over trials. We will include studies of at least two months.

Participants will be women diagnosed with PMS by prospective ratings of a validated scale (such as the Daily Symptoms Report^17^) or PMDD diagnosed by DSM or ICD criteria. We will exclude studies solely of women who self-report a diagnosis of PMS or PMDD, as this is known to be unreliable.^18^

The intervention will be luteal phase dosing (where the SSRI is taken only during the luteal phase of the cycle) or symptom onset dosing (where the SSRI is taken at first onset of premenstrual symptoms). We will exclude semi-intermittent dosing (where the SSRI is given at a higher dose during the luteal phase compared with the follicular phase).^19^ The comparison will be continuous dosing of an SSRI. We will include any SSRI: citalopram, escitalopram, fluoxetine, fluvoxamine, paroxetine, sertraline or zimelidine. We will also include venlafaxine and duloxetine; although these are not traditionally classified as SSRIs, their mechanism of action is similar at lower doses.

**Search Strategy**

The following databases will be searched from inception until December 2020: Cochrane Central Register of Controlled Trials, MEDLINE, EMBASE, PsycINFO, PubMed and CINAHL. References of previous reviews^14,15^ and included studies will also be searched. Two authors will screen study abstracts and retrieve potentially relevant full-texts for further examination. Disagreement will be resolved by discussion with a third author.

Examples of search keyword terms which will be combined are given below:

- Premenstrual Syndrome OR Premenstrual Dysphoric Disorder OR Late luteal OR PMS OR PMDD OR LLPDD
- Citalopram OR Escitalopram OR Fluoxetine OR Fluvoxamine OR Paroxetine OR Sertraline OR Zimelidine OR Duloxetine OR Venlafaxine
- Random OR Trial

**Data Extraction**

Two authors will extract data in duplicate with any disagreement discussed with a third author. The following variables of interest will be calculated from each study: study setting, sample size, mean age, diagnosis of interest, method of diagnosis, name of SSRI, dosage.

The two primary outcome measures will be response rate and dropout rate. A secondary outcome measure will be standardised mean difference of symptom ratings.

Response rate will be defined using as improvement on global scale (such as Clinical Global Impression Scale^20^) or as 50% reduction in symptoms using a continuous measure of symptoms score. Dropout rate will be defined as participants leaving the trial early for any reason. For studies reporting symptom scores, the standardised mean difference will be calculated between the intermittent and continuous dosing groups. Symptom end-scores will be extracted in preference to change scores. Where means and standard deviations are not reported, we will calculate an effect size based on reported sample size, test statistic and p values. We will analyse data on an intention-to-treat basis and request missing data from original study authors.

**Risk of Bias Assessment**

The Cochrane risk of bias assessment tool (version 2) will be used, independently by two authors, with disagreement resolved by a third author.

**Data Synthesis**

We will use a random-effects model to pool results across studies. Heterogeneity will be assessed using the I^2^ statistic. We will conduct subgroup analyses if more than three studies are available in the following categories: individual SSRI medication, dosage (low, medium or high), diagnosis (PMDD or PMS).

**REFERENCES**

1. Royal College of Psychiatrists. *Position Statement on Antidepressants and Depression*. Vol 49.; 2019. https://www.rcpsych.ac.uk/docs/default-source/improving-care/better-mh-policy/position-statements/ps04_19---antidepressants-and-depression.pdf?sfvrsn=ddea9473_5.

2. Davies J, Read J. A systematic review into the incidence, severity and duration of antidepressant withdrawal effects: Are guidelines evidence-based? *Addict Behav*. 2019. doi:10.1016/j.addbeh.2018.08.027

3. Jauhar S, Hayes J. The war on antidepressants: What we can, and can’t conclude, from the systematic review of antidepressant withdrawal effects by Davies and Read. *Addict Behav*. 2019. doi:10.1016/j.addbeh.2019.01.025

4. Hengartner MP. Commentary on Jauhar and Hayes. *Addict Behav*. 2019. doi:10.1016/j.addbeh.2019.02.007

5. Public Health England. Prescribed medicines review: summary. UK Government.

6. RCOG. Management of Premenstrual Syndrome: Green-top Guideline No. 48. *BJOG An Int J Obstet Gynaecol*. 2017;124(3):e73-e105. doi:10.1111/1471-0528.14260

7. Yonkers KA, Simoni MK. Premenstrual disorders. *Am J Obstet Gynecol*. 2018;218(1):68-74. doi:10.1016/j.ajog.2017.05.045

8. American Psychiatric Association. DSM-5 Diagnostic Classification. In: *Diagnostic and Statistical Manual of Mental Disorders*. ; 2013. doi:10.1176/appi.books.9780890425596.x00diagnosticclassification

9. Reed GM, First MB, Kogan CS, et al. Innovations and changes in the ICD-11 classification of mental, behavioural and neurodevelopmental disorders. *World Psychiatry*. 2019. doi:10.1002/wps.20611

10. Gehlert S, Song IH, Chang CH, Hartlage SA. The prevalence of premenstrual dysphoric disorder in a randomly selected group of urban and rural women. *Psychol Med*. 2009. doi:10.1017/S003329170800322X

11. Cohen LS, Soares CN, Otto MW, Sweeney BH, Liberman RF, Harlow BL. Prevalence and predictors of premenstrual dysphoric disorder (PMDD) in older premenopausal women: The Harvard study of moods and cycles. *J Affect Disord*. 2002. doi:10.1016/S0165-0327(01)00458-X

12. NICE. Depression in adults: recognition and management. *Natl Inst Heal Care Excell*. 2009.

13. Yonkers KA, Pearlstein T, Fayyad R, Gillespie JA. Luteal phase treatment of premenstrual dysphoric disorder improves symptoms that continue into the postmenstrual phase. *J Affect Disord*. 2005. doi:10.1016/j.jad.2004.10.006

14. Shah NR, Jones JB, Aperi J, Shemtov R, Karne A, Borenstein J. Selective serotonin reuptake inhibitors for premenstrual syndrome and premenstrual dysphoric disorder: A meta-analysis. *Obstet Gynecol*. 2008. doi:10.1097/AOG.0b013e31816fd73b

15. Marjoribanks J, Brown J, O’Brien PMS, Wyatt K. Selective serotonin reuptake inhibitors for premenstrual syndrome. *Cochrane Database Syst Rev*. 2013. doi:10.1002/14651858.CD001396.pub3

16. Moher D, Liberati A, Tetzlaff J, et al. Preferred reporting items for systematic reviews and meta-analyses: The PRISMA statement. *PLoS Med*. 2009. doi:10.1371/journal.pmed.1000097

17. Freeman EW, DeRubeis RJ, Rickels K. Reliability and validity of a daily diary for premenstrual syndrome. *Psychiatry Res*. 1996. doi:10.1016/S0165-1781(96)02929-0

18. Bosman RC, Albers CJ, De Jong J, Batalas N, Aan Het Rot M. No Menstrual Cyclicity in Mood and Interpersonal Behaviour in Nine Women with Self-Reported Premenstrual Syndrome. *Psychopathology*. 2018. doi:10.1159/000489268

19. Steiner M, Pearlstein T, Cohen LS, et al. Expert guidelines for the treatment of severe PMS, PMDD, and comorbidities: The role of SSRIs. *J Women’s Heal*. 2006. doi:10.1089/jwh.2006.15.57

20. Busner J, Targum SD. Global Impressions Scale : Applying a Research. *Psychiatry*. 2007.
